# Small Specimen Technology for Revealing Mechanical Properties of Alloys, 3D-Printing Metals and Welding Joints

**DOI:** 10.3390/ma16206648

**Published:** 2023-10-11

**Authors:** Jian Peng

**Affiliations:** 1 School of Mechanical Engineering and Rail Transit, Changzhou University, Changzhou 213164, China; jpeng@cczu.edu.cn; 2 Jiangsu Province Engineering Research Center of High-Level Energy and Power Equipment, Changzhou 213164, China

Small specimen technologies, such as the small punch test, the indentation test, and the in situ Scanning Electron Microscope (SEM)/Electron Back Scatter Diffraction (EBSD) test, provide important data support for understanding mechanical properties when the size of testing materials is limited, such as for alloys, 3D printing metals, and welding joints, as illustrated in Figure 1. They are developed to understand various mechanical properties, including tensile [1], creep [2], and fatigue properties [3], fracture parameters [4], hydrogen embrittlement [5], and stress corrosion cracks.

**Size scales and advantages of small specimen technologies:** For the small punch test, the specimen thickness size is usually 0.25 mm–0.5 mm. For the in situ SEM/EBSD test, a wide size range from μm to mm is feasible. For the indentation test, the specimen size is more flexible. Therefore, the development of small specimen technology gives us not only a new testing choice, but also a multiscale testing method. Different small specimen technologies have their own advantages. For the small punch test, the experimental equipment is easy to set up, while the mechanical properties of tensile, creep, and fatigue can be tested. The in situ SEM/EBSD test presents interesting microstructure developments when accompanied by mechanical testing. Both uncover mechanical behaviors, from elasticity and plasticity to failure fracture. For the indentation test, no additional sample needs to be taken from the studied materials, but only the elasticity and plasticity stages appear, while the failure fracture stage cannot be observed.

**Analyzing various mechanical properties using small specimen technologies:** Small specimen technologies are applied to analyze various mechanical properties, such as tensile, creep, fatigue, fracture, and environmental mechanical properties. The small punch test is used to predict the tensile strength parameters for numerous metals, while the small punch creep test can observe similar creep curves with different creep stages. Recently, they have also been applied to fatigue studies, and a novel setup with double punches was proposed [3]. Notched specimens are utilized to obtain fracture parameters such as *K*_IC_ and *J* integral. The combination of small specimen technology and a small environment container can ensure the feasibility and safety of mechanical tests in service environments, such as hydrogen. Small specimen technologies are developing into experienced experimental techniques for tensile and creep properties, but some complex mechanical properties need further verification and improvement, such as fatigue and fracture tests.

**Applications of small specimen technologies:** Small specimen technologies have been popular for a long time for serviced metals [6], 3D-printing metals [7], and welding joints [8]. They are of particular interest in engineering long-term service equipment as they provide advantages for micro or nondestructive sampling, which can be utilized to understand the damage degree of serviced metals. Three-dimensional-printing metals have great advantages in advanced manufacturing, but their mechanical properties are significantly different to those of traditional rolled metals, such as high anisotropy, weak fracture toughness, and specific creep and fatigue behaviors. At the same time, experimental data on mechanical properties for 3D-printing metals are lacking. Not only do standard large-sized specimens cost a lot for testing, but are also infeasible for in situ microstructure observations. Therefore, small specimen technologies are preferred for mechanical property studies on 3D-printing metals. Welding joints comprise a base metal zone, a heat-affected zone, and a melting zone, whose mechanical properties and failure modes are significantly different, while the traditional mechanical test with standard-size specimens only observes the mechanical property at the weakest position. Small specimen technologies make it easy to reveal the differences in mechanical properties at different welding joint positions.

**The correlation of small specimen technology with standard specimen test:** The testing results of small specimen technologies are different to those of the standard specimen test, especially for the small punch test and the indentation test, and correlation methods are needed. There are three kinds of correlation methods: empirical equations [9], theoretical derivation models [10], and machine learning models [11]. Empirical equations are very convenient in engineering applications, but the parameters in empirical equations are usually materials-dependent. The theoretical derivation model has physical significance, but the assumptions are unavoidable, which induces limited applications and un-negligible errors. Machine learning models are developed to correlate results through big data training, while physical meanings are usually lacking. A correlation method with both physical meaning and good prediction precision is urgently needed.

Although various small specimen technologies are developing quickly, there are still some knowledge ambiguities and gaps, such as the effect of size scale on mechanical properties; theoretically driven mechanical models of small specimen technologies; novel small specimen technologies of complex mechanical behaviors such as fatigue, creep–fatigue behavior, and fatigue crack propagation; fracture analyses; and data analysis methods. Moreover, systematic standards are urgently needed for engineering applications of small specimen technologies.

The current Special Issue concerns the latest developments in small specimen technologies, with particular attention to studies on the mechanical properties of alloys, 3D-printing metals, and welding joints. The scope of this Special Issue includes the following:

- Newly developed small specimen technology;
- Theoretical derivation mechanical models of small specimen technology;
- Comparison and correlation of small specimen technology with standard specimen tests;
- Applications of small specimen technology on advanced materials;
- Applications of small specimen technology on various mechanical properties, such as creep and fatigue;
- Fracture parameter analysis using notched or cracked small specimen technology;
- Small specimens under severe environments such as hydrogen and fused salt;
- Damage analyses of creep and fatigue behaviors using small specimen technology;
- Combination of machine learning methods and small specimen technology;
- Mechanical simulation and crystal plasticity simulation of small specimen technology;
- Reviews on small specimen technology.

## Figures and Tables

**Figure 1:**
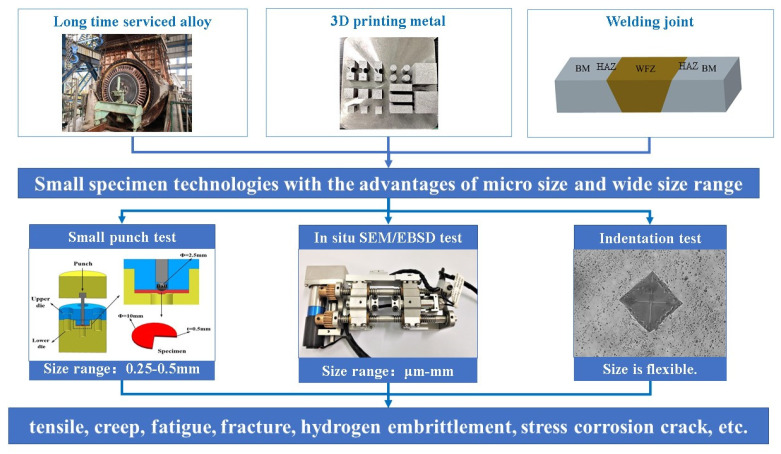
The development of small specimen technologies.
